# Structural tuning of photoluminescence in nanoporous anodic alumina by hard anodization in oxalic and malonic acids

**DOI:** 10.1186/1556-276X-7-228

**Published:** 2012-04-19

**Authors:** Abel Santos, Maria Alba, Mahbubur M Rahman, Pilar Formentín, Josep Ferré-Borrull, Josep Pallarès, Lluis F Marsal

**Affiliations:** 1 Departament d’Enginyeria Electrònica, Elèctrica i Automàtica, Universitat Rovira i Virgili, Avda. Països Catalans 26, Tarragona, 43007, Spain

**Keywords:** Photoluminescence, Hard anodization, Porous alumina, Pore widening, Heat treatment, Geometric characteristics

## Abstract

We report on an exhaustive and systematic study about the photoluminescent properties of nanoporous anodic alumina membranes fabricated by the one-step anodization process under hard conditions in oxalic and malonic acids. This optical property is analysed as a function of several parameters (i.e. hard anodization voltage, pore diameter, membrane thickness, annealing temperature and acid electrolyte). This analysis makes it possible to tune the photoluminescent behaviour at will simply by modifying the structural characteristics of these membranes. This structural tuning ability is of special interest in such fields as optoelectronics, in which an accurate design of the basic nanostructures (e.g. microcavities, resonators, filters, supports, etc.) yields the control over their optical properties and, thus, upon the performance of the nanodevices derived from them (biosensors, interferometers, selective filters, etc.)

## Background

Some applied sciences such as telecommunications, photocatalysis, photovoltaics, optoelectronics and biotechnology have registered a noticeable development throughout the last decades. One of the main reasons of that development is the better understanding over the optical properties of certain materials, among which nanoporous anodic alumina (NAA) is an outstanding example. Photoluminescence (PL) is the optical property by which a material absorbs photons of a given energy state from a source (i.e. it is excited) and emits photons of a lower energy state as a result of that excitation. Since the first study about the PL properties of NAA [1], many efforts have been made in order to understand the PL behaviour of this material [2-15]. Nowadays, NAA is a material commonly used in nanotechnology as a template or support for its versatility [16-22]. NAA is composed of hexagonally arranged nanometric pores in a matrix of alumina (Al_2_O_3_), the geometric characteristics of which (i.e. pore diameter, interpore distance, pore length, etc.) can be widely modified by changing the anodization conditions. NAA can be produced by means of several anodization strategies, the most representative examples of which are: i) the two-step anodization process under mild conditions (MA), by which NAA can be fabricated with 63, 100 and 500 nm interpore distances [23-28], ii) the one-step anodization process under hard conditions (HA), which makes it possible to produce NAA with interpore distances not attainable by MA (i.e. 220–300 nm) [29] and iii) the high-field anodization process (HFA), which expands the self-ordering regime of the two-step MA and the one-step HA (i.e. 300–450 nm) [6,30]. All this makes NAA a very versatile and cost-effective template for developing many nanostructures (e.g. nanowires, nanotubes, nanorods, etc.). Furthermore, as a consequence of its unique optical properties, NAA has demonstrated to be an excellent platform for developing such optical nanodevices as biosensors, interferometers, supports and so forth [31-35].

Presently, although the origin of the PL in NAA is still controversial, it is generally accepted that this relies on two types of photoluminescent centres namely, (1) *F*^*+*^ centres, which are related to the ionised oxygen vacancies [2,3] and (2) *F* centres associated with the carboxylate impurities from the acid electrolyte incorporated into the NAA structure during the anodization process [1]. The typical PL curve of NAA is a Gaussian-like bell with a maximum peak, the position and intensity of which depends on the quantity of *F* and *F*^*+*^ centres (i.e. increment of *F* centres → blue shift and PL increase and increment of *F*^*+*^ centres → red shift and PL increase). Previous studies have shown that the PL of NAA relies upon such fabrication parameters as the acid electrolyte, anodization voltage, pore diameter, thermal treatment, anodization regime and so forth [1-15]. The most commonly acid electrolytes used to fabricate NAA are sulphuric or H_2_SO_4_, oxalic (H_2_C_2_O_4_) and phosphoric (H_3_PO_4_) acids [28]. It has been observed that the PL intensity of NAA fabricated under MA conditions in oxalic is rather higher than that of NAA produced in sulphuric or phosphoric acids [8]. Furthermore, it has been found out that, for NAA produced in oxalic acid under MA, the PL intensity can be increased by an etch treatment (i.e. a pore widening) [1,9] or by a heat treatment [15]. Another question that is worth noting is that, for NAA fabricated in oxalic acid, the PL intensity is higher under MA than under HFA conditions. In addition, under these anodization regimes, the PL intensity increases as the hexagonal ordering improves [6,13]. More recently, some studies have reported about the PL properties of disordered NAA fabricated in some unusual acid electrolytes as sulfamic or H_3_SNO_3_ and malonic (H_4_C_3_O_4_) acids [10-12].

The success described in the studies just mentioned above necessitates that more exhaustive studies be carried out in order to understand and characterise completely the PL properties of NAA. Here, we present a complete and methodical study about the PL properties of nanoporous anodic alumina membranes (NAAMs) fabricated under hard anodization conditions in oxalic and malonic acids. These properties are studied as a function of several fabrication parameters (i.e. anodization voltage, pore diameter, membrane thickness, annealing temperature and acid electrolyte). This feature is a crucial factor for controlling the PL behaviour of NAAMs at will, what is dearly important for studying the PL properties of several nanostructures as nanowires or nanotubes by themselves (i.e. minimising or eliminating the PL interferences from the NAA template) [33,35] or designing active optical nanostructures as biosensors [31,32].

## Methods

### Aluminium pretreatment

Before anodizing, high-purity (99.999%) aluminium (Al) substrates from Goodfellow Cambridge Ltd. were electropolished in a mixture of ethanol and perchloric acid or HClO_4_ 4:1 (*v*:*v*) at 20 V. In this way, the surface roughness of the as-purchased Al substrates was reduced to nanometric scale. After this process, the Al substrates presented a mirror-like finished surface, what indicates that the surface was suitable for homogenous growth of NAA.

### Fabrication of nanoporous anodic alumina under hard anodization in oxalic acid

This type of NAAMs was fabricated following the one-step anodization process reported elsewhere [29]. In this process, the Al substrates were anodized in H_2_C_2_O_4_ 0.3 M at 0°C and 40 V for 10 min. Subsequently, the anodization voltage was increased at a rate of 0.1 V·s^-1^ until it reached the hard anodization voltage (i.e. 125, 130, 135 and 140 V). Then, the process was continued under that voltage till reaching the target membrane thickness ( Additional file 1: Figure S1a, b).

### Fabrication of nanoporous anodic alumina under hard anodization in malonic acid

These NAAMs were fabricated following a modified one-step anodization process [36]. First, the electropolished Al substrates were anodized in H_2_C_2_O_4_ 0.3 M at 0°C and 40 V for 10 min. Then, the anodization voltage was increased at a rate of 0.1 V·s^-1^ until it reached the hard anodization voltage (i.e. 125, 130, 135 and 140 V). Upon that, the process was continued under constant voltage for 5 min. Finally, the anodization process was maintained under the same conditions but replacing the acid electrolyte by malonic acid 1.7 M up to reach the membrane thickness established previously ( Additional file 1: Figure S1c, d).

### Pore opening and widening by wet chemical etching

After the anodization process, the remaining aluminium substrate was removed in a saturated solution of hydrochloric acid and cupric chloride or HCl/CuCl_2_. Subsequently, a pore opening process was performed by wet chemical etching in an aqueous solution of phosphoric acid 5 wt% at 35°C.

This procedure was carried out under current control, allowing us to control the pore opening with a high degree of accuracy [37,38]. Two examples of this process, one per each type of NAAM, are shown in Additional file 1: Figure S2. For those NAAMs used to analyse the effect of the pore widening time (i.e. the pore diameter) on the PL behaviour, a subsequent pore widening stage was performed by wet chemical etching in H_3_PO_4_ 5 wt% at 35°C.

### Nanoporous anodic alumina membranes characterization

The NAAMs were characterised by an environmental scanning electron microscope (ESEM FEI Quanta 600, Hillsboro, OR, USA). The elemental qualitative analysis of prepared NAAMs was carried out using energy dispersive X-ray spectroscopy (EDXS) coupled with the ESEM equipment. The photoluminescence measurements were taken on a fluorescence spectrophotometer from Photon Technology International Inc. (Birmingham, NJ, USA) with a Xe lamp used as the excitation light source at room temperature and an excitation wavelength (*λ*_*ex*_) of 350 nm. All the EDXS and PL measurements were performed on the HA side of the NAAMs (i.e. the bottom side). The standard image processing package (ImageJ, public domain programme developed at the Research Services Branch of the National Institutes of Health, USA) was used to carry out the ESEM image analysis [39].

## Results and discussion

To gain insight into the effect of each parameter (i.e. anodization voltage (*V*_HA_), pore diameter (*d*_p_) or pore widening time (*t*_PW_), membrane thickness (*τ*), annealing temperature (*T*) and acid electrolyte) on the PL behaviour of those NAAMs, their PL spectra were systematically and exhaustively analysed. To this end, the values of the experimental parameters were each fixed at *V*_HA_ = 140 V, *t*_PW_ = 0 min, *τ* = 50 μm and *T* = 100°C. When one of those parameters was modified to study its influence over the PL behaviour, the others remained fixed at those values.

**Figure 1:**
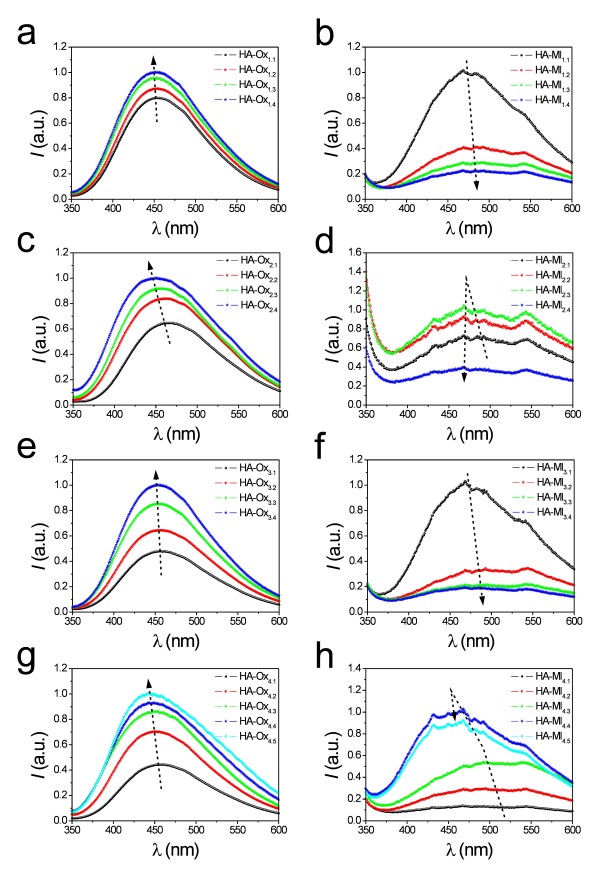
**(a)** HA-Ox_1.1_-HA-Ox_1.4_. **(b)** HA-Ml_1.1_-HA-Ml_1.4_. **(c)** HA-Ox_2.1_-HA-Ox_2.4_. **(d)** HA-Ml_2.1_-HA-Ml_2.4_. **(e)** HA-Ox_3.1_-HA-Ox_3.4_. **(f)** HA-Ml_3.1_-HA-Ml_3.4_. **(g)** HA-Ox_4.1_-HA-Ox_4.5_. **(h)** HA-Ml_4.1_-HA-Ml_4.5_.

Figure 1 shows the PL spectra of all these NAAMs, the characteristics of which are summarised in Table 1. At first glance, it is observed that the PL spectra of those NAAMs are rather wide and asymmetric, which denotes the presence of several luminescent centres (i.e. *F*^*+*^ and *F*).

**Table 1:** Geometric characteristics and fabrication parameters of the NAAMs fabricated in oxalic and malonic acids

**Label**	**Parameter**	**Value**
HA-Ox_1.1_ and HA-Ml_1.1_	*V*_*HA*_ (Volts)	125
HA-Ox_1.2_ and HA-Ml_1.2_	“	130
HA-Ox_1.3_ and HA-Ml_1.3_	“	135
HA-Ox_1.4_ and HA-Ml_1.4_	“	140
HA-Ox_2.1_ and HA-Ml_2.1_	*t*_*PW*_ (minute) (*d*_*p*_ (nanometer))	0 (64 ± 6) and 0 (64 ± 7)
HA-Ox_2.2_ and HA-Ml_2.2_	“	15 (101 ± 7) and 5 (87 ± 7)
HA-Ox_2.3_ and HA-Ml_2.3_	“	30 (122 ± 9) and 10 (111 ± 8)
HA-Ox_2.4_ and HA-Ml_2.4_	“	45 (150 ± 9) and 15 (116 ± 7)
HA-Ox_3.1_ and HA-Ml_3.1_	*τ*micrometer	20
HA-Ox_3.2_ and HA-Ml_3.2_	“	30
HA-Ox_3.3_ and HA-Ml_3.3_	“	40
HA-Ox_3.4_ and HA-Ml_3.4_	“	50
HA-Ox_4.1_ and HA-Ml_4.1_	*T* (degrees Celsius)	100
HA-Ox_4.2_ and HA-Ml_4.2_	“	200
HA-Ox_4.3_ and HA-Ml_4.3_	“	300
HA-Ox_4.4_ and HA-Ml_4.4_	“	400
HA-Ox_4.5_ and HA-Ml_4.5_	“	500

**Figure 2:**
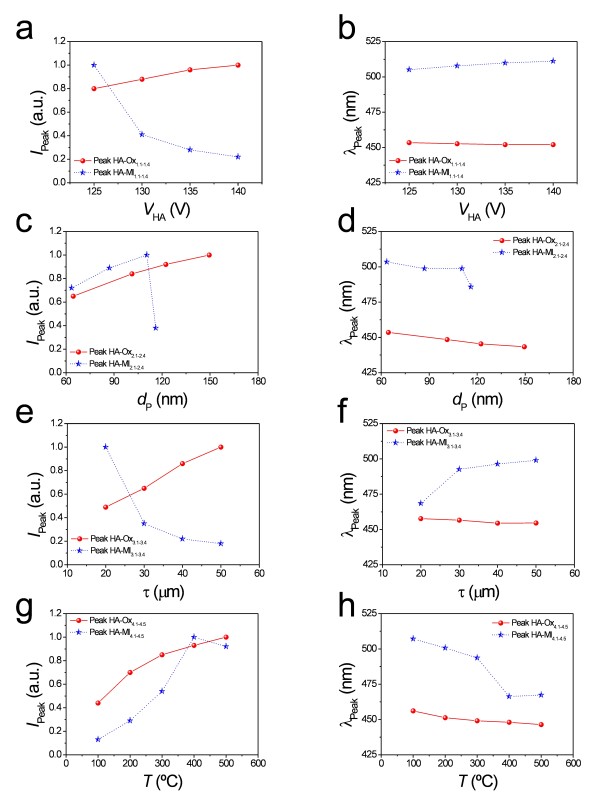
**(a)** and **(b)** HA-Ox_1.1_-HA-Ox_1.4_ and HA-Ml_1.1_-HA-Ml_1.4_. **(c)** and **(d)** HA-Ox_2.1_-HA-Ox_2.4_ and HA-Ml_2.1_-HA-Ml_2.4_. **(e)** and **(f)** HA-Ox_3.1_-HA-Ox_3.4_ and HA-Ml_3.1_-HA-Ml_3.4_. **(g)** and (h) HA-Ox_4.1_-HA-Ox_4.5_ and HA-Ml_4.1_-HA-Ml_4.5_.

To analyse the effect of the experimental parameters on the PL behaviour, the wavelength and intensity of the peaks in the PL spectra were represented versus each parameter (Figure 2). In general, it was observed that the influence of the experimental parameters over the intensity of those subpeaks was noticeable. However, the subpeak positions were rather stable in all the cases.

Depending on the acid electrolyte, the NAAMs were labelled as HA-Ox_*i.j*_ for oxalic acid and HA-Ml_*i.j*_ for malonic acid, where the subscripts *i* and *j* denote the parameter analysed (i.e. 1 = hard anodization voltage, 2 = pore widening time, 3 = membrane thickness and 4 = annealing temperature) and the value of that parameter (i.e. 1 = as-produced value and 4 or 5 = last value of that set), respectively.

### Hard anodization voltage

Figure 1a shows the PL spectra of samples HA-Ox_1.1_ to HA-Ox_1.4_. From this, it is verified that there is a slight linear increase of the PL peak intensity as the hard anodization voltage increases from 125 to 140 V (Figure 2a). The peak position remains nearly constant as the hard anodization voltage increases (Figure 2b).

The PL behaviour of samples HA-Ml_1.1_ to HA-Ml_1.4_ differs from that of those NAAMs fabricated in oxalic acid under the same anodization conditions (Figure 1b). The intensity of the PL peak decreases exponentially as *V*_HA_ increases from 125 to 140 V (Figure 2a), and there is a slight red shift of the peak position throughout the anodization voltage increase (Figure 2b).

As was commented above, the origin of the photoluminescence in NAA fabricated in oxalic and malonic acid is attributed to *F*^*+*^ centres and *F* centres [1-15]. It was confirmed by EDXS analysis ( Additional file 1: Figure S3 and Table S1) that the content of carboxylate impurities of NAA fabricated in oxalic and malonic acids under hard anodization decreases as *V*_HA_ increases, which is in good agreement with previous studies [29]. These impurities are mainly *F* centres, which disturb the transmittance of the light from the excitation source as well as the light emission from the oxygen vacancies (i.e. *F*^*+*^ centres) [1].

For those samples fabricated in oxalic acid, the increase of the PL peak intensity with *V*_HA_ can be related to a decrease in the carboxylate impurities in the NAA structure, although this does not produce a noticeable change in the PL peak position. That behaviour is rather different for those samples fabricated in malonic acid under the same anodization conditions. The exponential decrease of the PL peak intensity with the hard anodization voltage indicates that the carboxylate impurities in the NAA structure decrease with *V*_HA_, as demonstrated by EDXS analysis too ( Additional file 1: Figure S3 and Table S1). However, the red shift observed in the PL peak position means that the oxygen evolution as well as the tensile stress and the electrostriction pressure in the NAA structure increase as *V*_HA_ increases [40].

### Pore widening time – pore diameter

**Figure 3:**
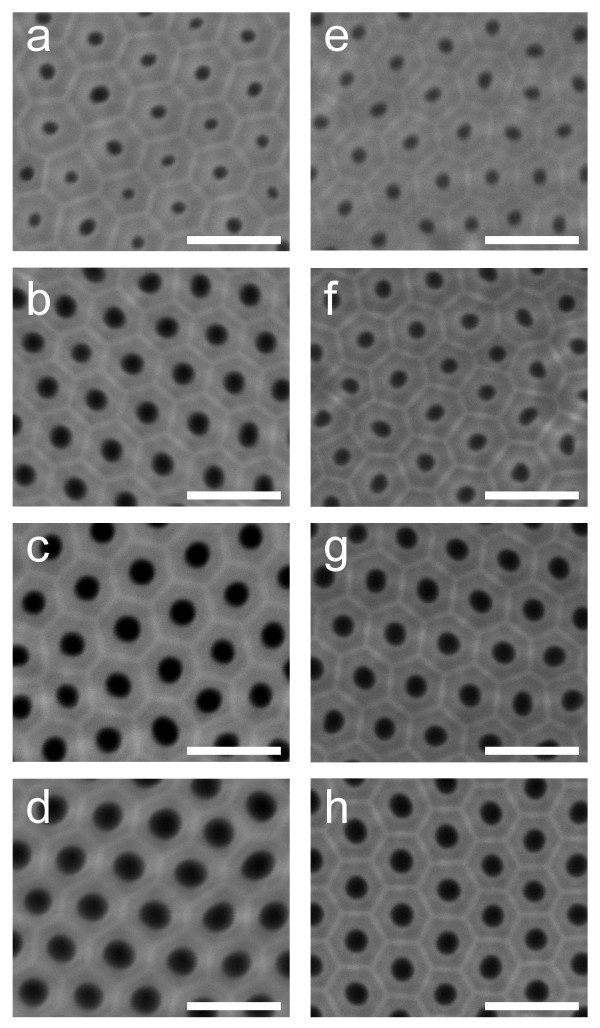
**(a)** HA-Ox_2.1_ (*t*_*PW*_ = 0 min). **(b)** HA-Ox_2.2_ (*t*_*PW*_ = 15 min). **(c)** HA-Ox_2.3_ (*t*_*PW*_ = 30 min). **(d)** HA-Ox_2.4_ (*t*_*PW*_ = 45 min). **(e)** HA-Ml_2.1_ (*t*_*PW*_ = 0 min). **(f)** HA-Ml_2.2_ (*t*_*PW*_ = 5 min). **(g)** HA-Ml_2.3_ (*t*_*PW*_ = 10 min). (h) HA-Ml_2.4_ (*t*_*PW*_ = 15 min).

The HA side of those NAAMs used to analyse the pore diameter effect on the PL properties was inspected by ESEM after the pore opening and widening stages (Figure 3). The average pore diameter was obtained from ESEM image analysis (Table 1).

Figure 1c shows the PL spectra of samples HA-Ox_2.1_ to HA-Ox_2.4_. As a first result, it is observed that there is a noticeable linear increase of the PL peak intensity with the pore diameter (Figure 2c). Moreover, there is a slight blue shift in the PL peak position as *d*_p_ is enlarged (Figure 2d).

Figure 1d represents the PL spectra of samples HA-Ml_2.1_ to HA-Ml_2.4_. It shows that there is a linear increase of the PL peak intensity until 10 min of pore widening and an abrupt decrease between 10 and 15 min (Figure 2c). Furthermore, there is a slight blue shift of the peak position up to 10 min, which gets more marked from 10 to 15 min (Figure 2d).

**Figure 4:**
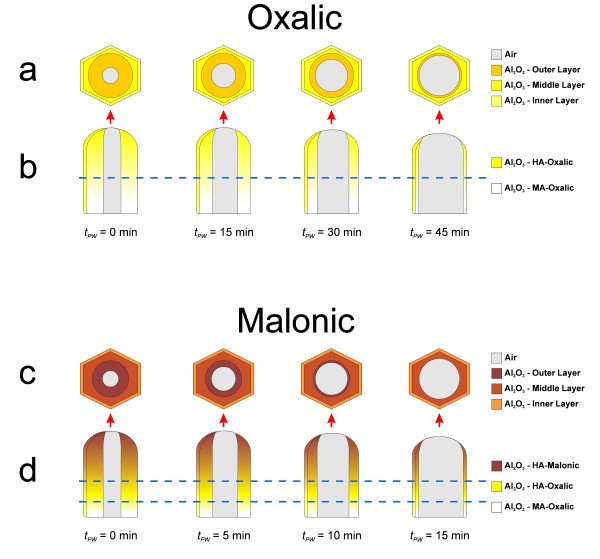
Set of illustrations of the pore structure as a function of the pore widening time in nanoporous anodic alumina fabricated in oxalic ((a) top and (b) cross-section views) and in malonic acids ((c) top and (d) cross-section views).

To understand the pore widening effect on the PL spectrum of those NAAMs, it is necessary to point out that the pore wall structure in NAA can be divided into three layers (Figure 4) namely, (1) outer layer (*F* centres from electrolyte impurities), (2) middle layer (*F*^*+*^ centres from oxygen vacancies) and (3) inner layer (pure Al_2_O_3_ without PL centres). As was previously commented, the outer layer disturbs the light transmittance from the excitation source as well as the light emission from the middle layer [1]. During the pore widening process, the outer layer is progressively dissolved and, thus, the PL intensity increases since the light transmittance from the source and the light emission from the middle layer are less hindered by the electrolyte impurities. However, when the outer layer is entirely dissolved, the acid solution starts to dissolve the middle layer and the PL intensity decreases. Hence, the limit of each layer can be established by means of the PL analysis. Concerning to the peak position, there is a blue shift because the *F* centres distributed through the outer layer are progressively removed during the etching process. Furthermore, this blue shift becomes more noticeable when the middle layer is reached owing to the main portion of *F*^*+*^ centres is concentrated in this layer.

As Figure 1c shows, for samples HA-Ox_2.1_ to HA-Ox_2.4_, the pore widening process produces a linear increase of the PL peak intensity from 0 to 45 min of pore widening time. These results agree with previous studies, which reported about the PL dependence on the pore widening time in NAA fabricated in oxalic acid under MA conditions [9]. However, according to other studies on the same type of NAA, it is expected that the PL peak intensity decreases for longer etching times [1]. So, it is possible to deduce that after the pore widening process (i.e. Table 1*t*_PW_ = 45 min → *d*_*p*_ = 150 ± 9 nm), the middle layer of the pore walls has not been reached yet. Otherwise, a decrease in the PL peak intensity would have been observed.

In the case of samples HA-Ml_2.1_ to HA-Ml_2.4_, there is a noticeable increase in the PL intensity up to 10 min of pore widening (Figure 1d). Nevertheless, for longer etching times (i.e. 15 min), the PL peak intensity decreases sharply. This means that the middle layer of the pore walls is partially dissolved during this etching time. This result certifies that the outer layer is thinner than that of NAAMs produced in oxalic acid (i.e. Table 1*t*_PW_ = 15 min → *d*_p_ = 116 ± 7 nm).

Another interesting result is that for the same NAAM geometry (i.e. thickness, interpore distance and pore diameter), the etching rate is much faster in those NAAMs fabricated in malonic acid. Preliminary experiments verified that the NAAMs fabricated in malonic acid collapsed after 30 min of pore widening under these etching conditions ( Additional file 1: Figure S4).

### Membrane thickness

Figure 1e shows the PL spectra of samples HA-Ox_3.1_ to HA-Ox_3.4_. At first sight, it is seen that the thicker the NAAM, the higher the PL peak intensity (Figure 2e). Additionally, the PL peak position is fairly stable with the membrane thickness increase (Figure 2f). These results agree with previous studies about the effect of the membrane thickness on the PL of NAA fabricated in oxalic acid under MA conditions [5,7].

The PL spectra of samples HA-Ml_3.1_ to HA-Ml_3.4_ are summarised in Figure 1f. In contrast to those samples fabricated in oxalic acid, it is clear that the thicker the NAAM the lower the PL peak intensity (Figure 2e). Regarding the PL peak position, there is a noticeable red shift when the membrane thickness is increased from 20 to 30 μm and it becomes nearly constant from 30 to 50 μm (Figure 2f).

To interpret the effect of the membrane thickness on the PL behaviour, it is necessary to know the membrane structure ( Additional file 1: Figure S1a, c). The cross sectional structure of NAAMs fabricated in oxalic acid can be divided into two main layers namely, (1) a thin protective oxide layer of about 500 nm with disordered pores and (2) a thick oxide layer with ordered pores, the thickness of which depends on the target thickness (i.e. about 20, 30, 40 or 50 μm). These layers are fabricated under MA and HA conditions, respectively, which endows them with different optical properties (e.g. photoluminescence) [29]. Nevertheless, bearing in mind that the HA layer is about 100 times thicker than the MA layer, the effect of the protective oxide layer on the PL properties of this type of NAAMs can be considered negligible. The structure of NAAMs produced in malonic acid is constituted of three main layers namely, (1) a thin protective oxide layer of about 500 nm with disordered pores, (2) an intermediate oxide layer of approximately 5 μm with ordered pores and (3) a thick layer with ordered pores, the thickness of which depends on the target thickness (i.e. approximately 15, 25, 35 and 45 μm for a total thickness of 20, 30, 40 and 50 μm, respectively). The first and second layers are fabricated in oxalic acid under MA and HA conditions, respectively. The optical properties of these layers are different although the effect of the protective oxide layer on the whole PL behaviour of the NAAM can be neglected. Nonetheless, when the thickness of the malonic layer is thinner or equal to 15 μm (i.e. sample HA-Ml_3.1_), there is a noticeable influence of the intermediate oxide layer on the PL spectrum. For this reason, the PL peak intensity decreases with the thickness of the malonic layer. That behaviour would denote that somehow the malonic layer produces a screen effect on the emission from the intermediate layer generated in oxalic acid, the PL intensity of which is higher than that of the malonic layer. This screen effect is more efficient as the thickness of the malonic layer increases and is reflected in the peak position as well. As we can see in Figure 2f, at 20 μm the peak position of both types of membranes is rather similar since the thickness of the malonic layer is still thin. However, as the thickness of the malonic layer is increased, a marked red shift is produced owing to the PL peak position of the NAA fabricated in malonic acid are located at longer wavelengths.

### Annealing temperature

The PL spectra of samples HA-Ox_4.1_ to HA-Ox_4.5_ are summarised in Figure 1g. The relationships between the PL peak intensity and its position and the annealing temperature are represented in Figures 2g,h, respectively. Notice that there is an exponential increase of the PL peak intensity when *T* is increased from 100°C to 500°C. Concerning to the PL peak position, there is a slight blue shift throughout the annealing treatment, which agrees with previous studies [6,11].

Figure 1h shows the PL spectra of samples HA-Ml_4.1_ to HA-Ml_4.5_. The PL peak intensity increases linearly with the annealing temperature until 400°C, and there is a moderate decrease at higher temperatures (i.e. from 400°C to 500°C) (Figure 2g). As for the PL peak position, there is a marked blue shift from 100°C to 300°C, which gets more prominent from 300°C to 400°C. Finally, from 400°C to 500°C, the PL peak position remains almost stable (Figure 2h).

It was verified that the effect of the annealing temperature on the PL behaviour of those NAAMs fabricated in oxalic acid under HA is rather similar to that of NAA fabricated under MA and HFA [6,11]. The increase of the PL peak intensity with the annealing temperature can be related to the thermal decomposition of carboxylate impurities located at the pore surface (i.e. outer layer) [11,15].

The annealing effect on the PL properties of those NAAMs fabricated in malonic acid under HA conditions differs slightly from those fabricated under MA [11,12] because no relative minimum of the PL peak intensity is observed at intermediate temperatures (i.e. from 200°C to 300°C) [11]. In addition, the maximum intensity is reached when the annealing temperature is about 400°C which is a lower temperature than that of NAA fabricated under MA conditions in the same acid electrolyte (i.e. about 600°C) [11,12].

It is known that the initial increase in the PL peak intensity of NAA fabricated in malonic and oxalic acid as a result of an annealing treatment is associated with the decomposition of the malonic and oxalic impurities located at the pore surface from 100°C to 400°C. The subsequent PL decrease is related to a decrease in the oxygen vacancies as a result of the crystallographic phase change in the NAA structure as well as the decomposition of the malonic and oxalic impurities distributed through the NAA bulk. This process starts between 400°C and 500°C for NAAMs fabricated in malonic acid and at higher temperatures (i.e. around 500°C) for those produced in oxalic acid [4]. In both types of NAAMs, the PL peak is shifted towards shorter wavelengths (i.e. blue shift) as a result of the decomposition of superficial impurities at low temperatures, which allows increase the light emission from the outer layer. This shift is more remarkable for those NAAMs produced in malonic acid since the malonic impurities decompose at lower temperatures. Furthermore, there is a slight red shift of the PL peak position at higher temperatures which is associated with the decomposition of the electrolyte impurities distributed through the bulk of the outer layer [4].

### Acid electrolyte

**Figure 5:**
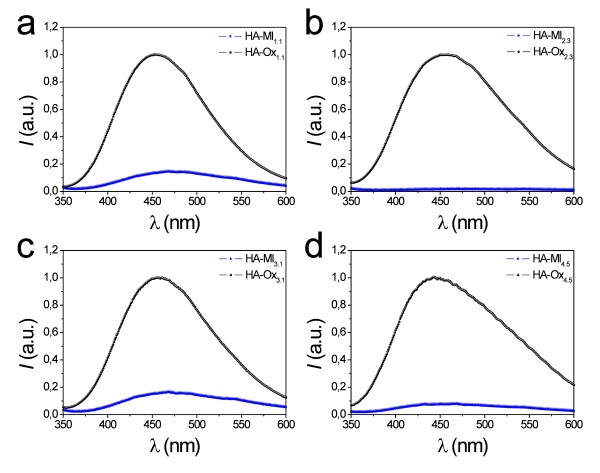
**(a)** HA-Ox_1.1_ and HA-Ml_1.1_. **(b)** HA-Ox_2.3_ and HA-Ml_2.3_. **(c)** HA-Ox_3.1_ and HA-Ml_3.1_. **(d)** HA-Ox_4.5_ and HA-Ml_4.5_.

To correctly compare the PL properties of those NAAMs fabricated in different acid electrolytes, the interpore distance and the pore diameter were each set to 280 and 64 nm, approximately (i.e. *V*_*an*_ = 140 V and *t*_*PW*_ = 0 min, respectively). Figure 5 shows a comparison between the PL spectra of some nanoporous anodic alumina membranes fabricated in oxalic and malonic acids at different fabrication conditions. From this, it is patent that the PL peak intensity of those NAAMs fabricated in oxalic acid under HA conditions is higher than that of those fabricated in malonic acid under the same anodization conditions. This difference in the PL intensity could be attributed to three main structural characteristics of the resulting NAA namely, (1) the carbon content (i.e. carboxylate impurities), (2) the thickness of the outer layer in the pore structure and (3) the quantity of oxygen vacancies in the NAA structure.

It was verified by EDXS analysis that the carbon content (i.e. carboxylate impurities) is higher in those NAAMs fabricated in malonic acid ( Additional file 1: Figure S3). This can be observed also by a noticeable change in colour between NAAMs fabricated in oxalic (yellow) and malonic (bright brown) acids ( Additional file 1: Figure S4). Another question is that, as was demonstrated in section 3.2, the outer layer thickness (i.e. layer with carboxylate impurities) is thinner for those NAAMs produced in malonic acid. Therefore, the concentration of carboxylate impurities is rather higher in these NAAMs (i.e. thinner outer layer with more carboxylate impurities). These two factors imply that the screen effect of the outer layer is more effective in NAAMs produced in malonic acid (i.e. higher disturbance of the light transmittance from the excitation source and the light emission from the oxygen vacancies) [1]. In addition, the oxygen vacancies in the structure of NAAMs fabricated in malonic acid are lower than that of those produced in oxalic acid because of the reduction in the oxygen evolution, tensile stress and electrostriction pressure at high anodization voltage.

## Conclusions

In conclusion, we have presented an exhaustive and systematic study on the photoluminescent properties of nanoporous anodic alumina membranes fabricated by the one-step anodization process under hard conditions in oxalic and malonic acids. The PL properties of these membranes have been analysed as a function of several experimental parameters (i.e. hard anodization voltage, pore widening time, anodization time, annealing temperature and acid electrolyte). It has been found out that the outer layer of the pore structure in NAAMs fabricated in malonic acid is thinner than that of NAAMs produced in oxalic acid, although the PL absorption of this layer is more effective in the first type of NAAMs because of the higher concentration of carboxylate impurities. Furthermore, it has been inferred that the quantity of oxygen vacancies is lower in NAAMs produced in malonic acid.

All this demonstrates that the PL properties of NAAMs fabricated in oxalic and malonic acids are not only related to their geometric characteristics but also on the acid electrolyte. Likewise, depending on the acid electrolyte, these relationships are ostensibly different. Therefore, to establish and understand the PL reliance on these factors is crucial for designing and developing nanostructures and nanodevices with well-controlled optical properties.

## Competing interest

The authors AS, MA, MMR, PF, JFB, JP and LFM declare that they have no competing interests.

## Authors’ contributions

The experiments presented in this work were designed by AS and LFM. The NAAMs were fabricated by AS, MA and MMR, characterised optically by AS, MA and MMR, and the ESEM analysis was performed by AS and MA. PF assisted AS, MA and MMR with the laboratory tasks. AS, MA, PF, JFB, JP and LFM analysed and discussed the results obtained from the experiments. AS wrote the manuscript and the last version of this was read and approved by all the authors (AS, MA, MMR, PF, JFB, JP and LFM).

## Supplementary Material

Additional file 1 **Table S1** Average percentage content of Aluminium (Al), Oxygen (O) and carbon (C) of samples HA-Ox1.1-HA-Ox1.4 and HA-Ml1.1-HA-Ml1.4 after EDXS analysis. The elemental qualitative analysis was performed on the HA side of each NAAM at three different areas. **Figure S1.** Illustration of the resulting nanoporous anodic alumina structure and the current density and voltage-time transients (i.e. *J*-*t* and *V-t*) corresponding to the one-step hard anodization in oxalic and malonic acids. (**a**) and (**b**) oxalic acid. (**c**) and (**d**) malonic acid. **Figure S2.** Voltage and current density-time (*J*-*t*) transient recorded during the pore opening process and illustrations of the pore structure related to each stage of this process for a NAAM fabricated in oxalic ((**a**) and (**b**)) and in malonic ((**c**) and (**d**)) acids. **Figure S3.** Carbon content of samples HA-Ox1.1-HA-Ox1.4 and HA-Ml1.1-HA-Ml1.4 as a function of the hard anodization voltage (*VHA*) after EDXS analysis. **Figure S4.** Collapse of NAAMs fabricated in malonic acid after 30 ((a)) and 45 ((b)) min of pore widening in H3PO4 5 wt% at 35ºC (*scale bar* = 2 μm). **Figure S5.** Digital photography of two NAAMs fabricated in oxalic and malonic acid (*scale bar* = 20 mm) Click here for file
