# Supplementary material for: Structural tuning of photoluminescence in nanoporous anodic alumina by hard anodization in oxalic and malonic acids
Source: Nanoscale Res Lett. 2012 Apr 19;7(1):228. doi: 10.1186/1556-276X-7-228 (PMC3413565; doi:10.1186/1556-276X-7-228)
Supplement: Additional file 1 — Table S1 Average percentage content of Aluminium (Al), Oxygen (O) and carbon (C) of samples HA-Ox1.1-HA-Ox1.4 and HA-Ml1.1-HA-Ml1.4 after EDXS analysis. The elemental qualitative analysis was performed on the HA side of each NAAM at three different areas. Figure S1. Illustration of the resulting nanoporous anodic alumina structure and the current density and voltage-time transients (i.e. J-t and V-t) corresponding to the one-step hard anodization in oxalic and malonic acids. (a) and (b) oxalic acid. (c) and (d) malonic acid. Figure S2. Voltage and current density-time (J-t) transient recorded during the pore opening process and illustrations of the pore structure related to each stage of this process for a NAAM fabricated in oxalic ((a) and (b)) and in malonic ((c) and (d)) acids. Figure S3. Carbon content of samples HA-Ox1.1-HA-Ox1.4 and HA-Ml1.1-HA-Ml1.4 as a function of the hard anodization voltage (VHA) after EDXS analysis. Figure S4. Collapse of NAAMs fabricated in malonic acid after 30 ((a)) and 45 ((b)) min of pore widening in H3PO4 5 wt% at 35ºC (scale bar = 2 μm). Figure S5. Digital photography of two NAAMs fabricated in oxalic and malonic acid (scale bar = 20 mm) [file 1556-276X-7-228-S1.pdf]

Supporting Information

Structural Tuning of Photoluminescence in Nanoporous Anodic Alumina by Hard Anodization in Oxalic and Malonic Acids

Abel Santos, María Alba, Mohammad Mahbub Rahman, Pilar Formentín, Josep Ferré-Borrull, Josep Pallarès and Lluís F. Marsal[1]

Departament d'Enginyeria Electrònica, Elèctrica i Automàtica
Universitat Rovira i Virgili
Avda. Països Catalans 26 - 43007 Tarragona-Spain

[1] To whom correspondence should be addressed (e-mail: lluis.marsal@urv.cat)

S.1. Hard Anodization

The one-step anodization for fabricating nanoporous anodic alumina (NAA) under hard anodization conditions is divided into three stages (**Scheme S1**). First, when the acid electrolyte temperature is around 0ºC, the anodization process starts under constant voltage at 40 V. After this, the anodization voltage is increased at a rate of 1 $V{\cdot}s^{-1}$ until it reaches the target voltage (i.e. hard anodization voltage). Then, the anodization voltage is maintained constant until the desired NAA thickness is reached. **Schemes S1b and d** show a typical current density-time (*J*-*t*) transient of a one-step anodization process under hard anodization conditions at 140 V in oxalic and malonic acids, respectively. During the one-step anodization process, the following three different anodization regimes take place:

i) Mild anodization (MA) regime at 40 V.
ii) Transition anodization (TA) regime at voltage ramp 1 $V{\cdot}s^{-1}$.
iii) Hard anodization (HA) regime at 140 V.

These different anodization regimes (i.e. MA, TA and HA) generate three different layers on the resulting NAA. During the first stage, a protective layer of NAA with disordered pores is formed on the aluminium surface. This layer is about 500 nm thick and has two main functions: namely, i) to suppress breakdown effects due to high temperatures and ii) to enable uniform oxide film growth at high voltage. When the anodization voltage is increased, the pores are reorganized because the anodization conditions modify the interpore distance. In this way, some pores vanish and others continue growing by the self-ordering mechanism during the transition regime.

Finally, when the hard anodization voltage is reached, the pores growth uniformly at an exponential growth rate, which is characteristic of a hard anodization process. The result is a NAA film with disordered and ordered pores on the top and bottom sides, respectively (**Schemes S1a and c**).

S.2. Pore Opening by Current Control

The pore opening process was performed in a home-made electrochemical cell based on two containers connected each other by two tubes. The NAAMs were placed between these tubes with the top side (i.e. open pores) facing the container 1 and the bottom side (i.e. closed pores) facing the container 2. The container 1 was filled with an aqueous solution of KCl 0.2 M and the container 2 with H_3PO_4 5 wt%. Both solutions were constantly stirred at 35 ºC. Two stainless steel electrodes immersed in the containers were connected to a power supply and a voltage of 2 V was applied between them. The current density (J) was monitored during the etching process. As **Scheme S2** shows, the current density transient can be divided into three stages, which correspond to different stages of the pore opening process: namely, i) t_1 – J remains relatively constant, what denotes that the oxide barrier layer at the pore bottom tips is compact, ii) t_2 – there is a noticeable and progressive increase in J as a result of the ion permeation through some open pores and iii) t_3 – J becomes steady again but at a higher value because of the ion transport takes place through all the pores → Pore Opening Point. It was verified that the etching rate was always faster for those NAAMs fabricated in malonic than in oxalic acid.

S.3. Elemental Qualitative Analysis

An elemental qualitative analysis of some NAAMs (i.e. HA-$Ox_{1.1}$-HA-$Ox_{1.4}$ and HA-$Ml_{1.1}$-HA-$Ml_{1.4}$) was performed in order to estimate the effect of the carbon content on the PL behaviour. This analysis was carried out using energy dispersive X-ray spectroscopy (EDXS) coupled with the ESEM equipment. The results are summarized in **Figure S1** and **Table S1**.

S.4. Collapse of NAAMs by Excessive Pore Widening

From preliminary experiments, it was established that the total collapse of the NAAM structure of those samples fabricated in malonic acid takes place after 30 min of wet chemical etching in H_3PO_4 5 wt% at 35ºC. **Figure S2** shows the collapsed structure of two NAAMs after 30 (**Figure S2a**) and 45 min (**Figure S2b**) of pore widening.

S.5. Difference in Colour

It was observed with the naked eye that those NAAMs fabricated in oxalic acid presented a yellow colour and those produced in malonic acid were bright brown (**Figure S3**). It is deduced that such change in colour can be related to the carbon content. This matter was verified by EDXS analysis.

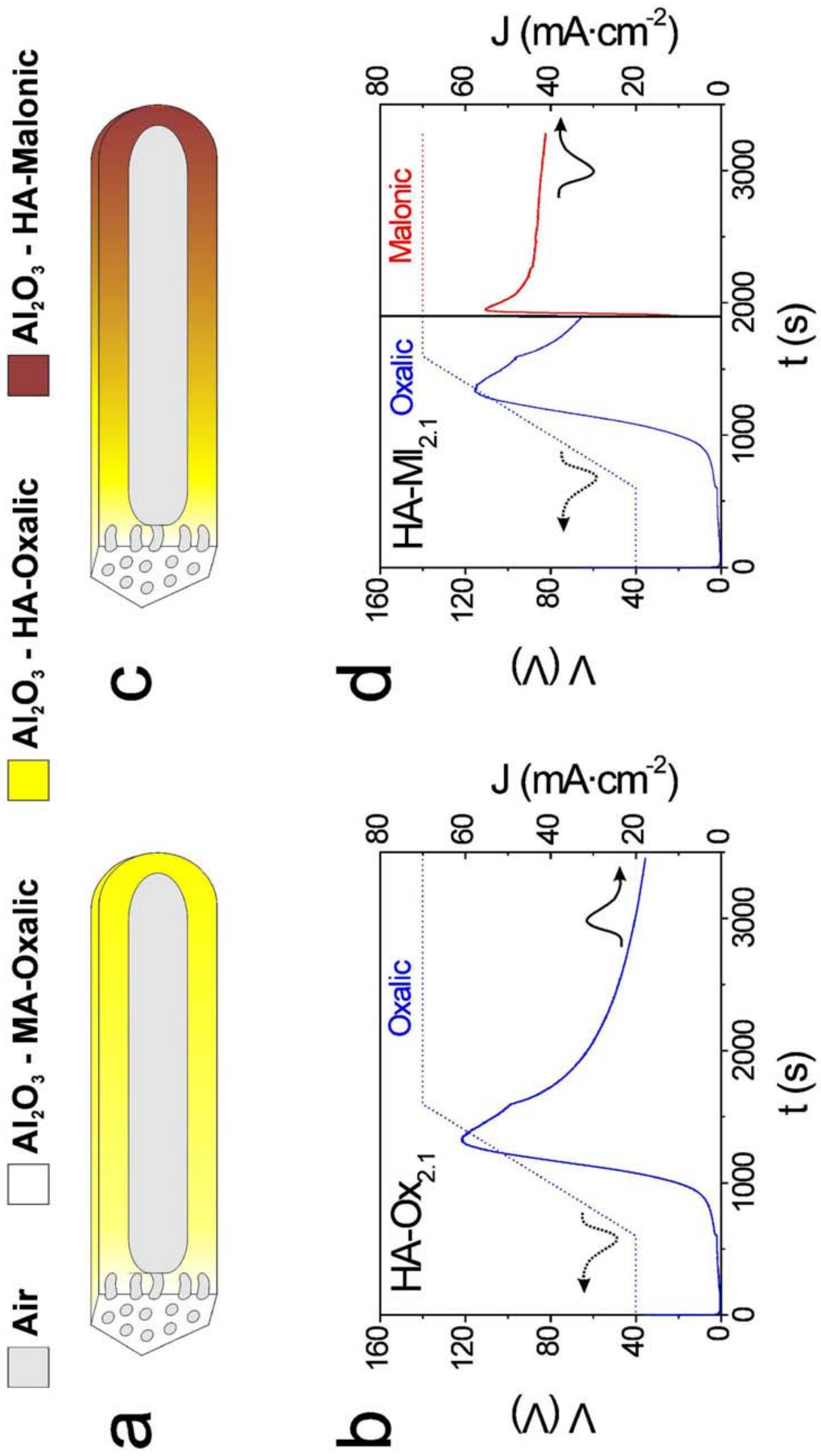

Scheme S1 Illustration of the resulting nanoporous anodic alumina structure and the current density and voltage-time transients (i.e. *J-t* and *V-t*) corresponding to the one-step hard anodization in oxalic and malonic acids. (a) and (b) oxalic acid. (c) and (d) malonic acid.

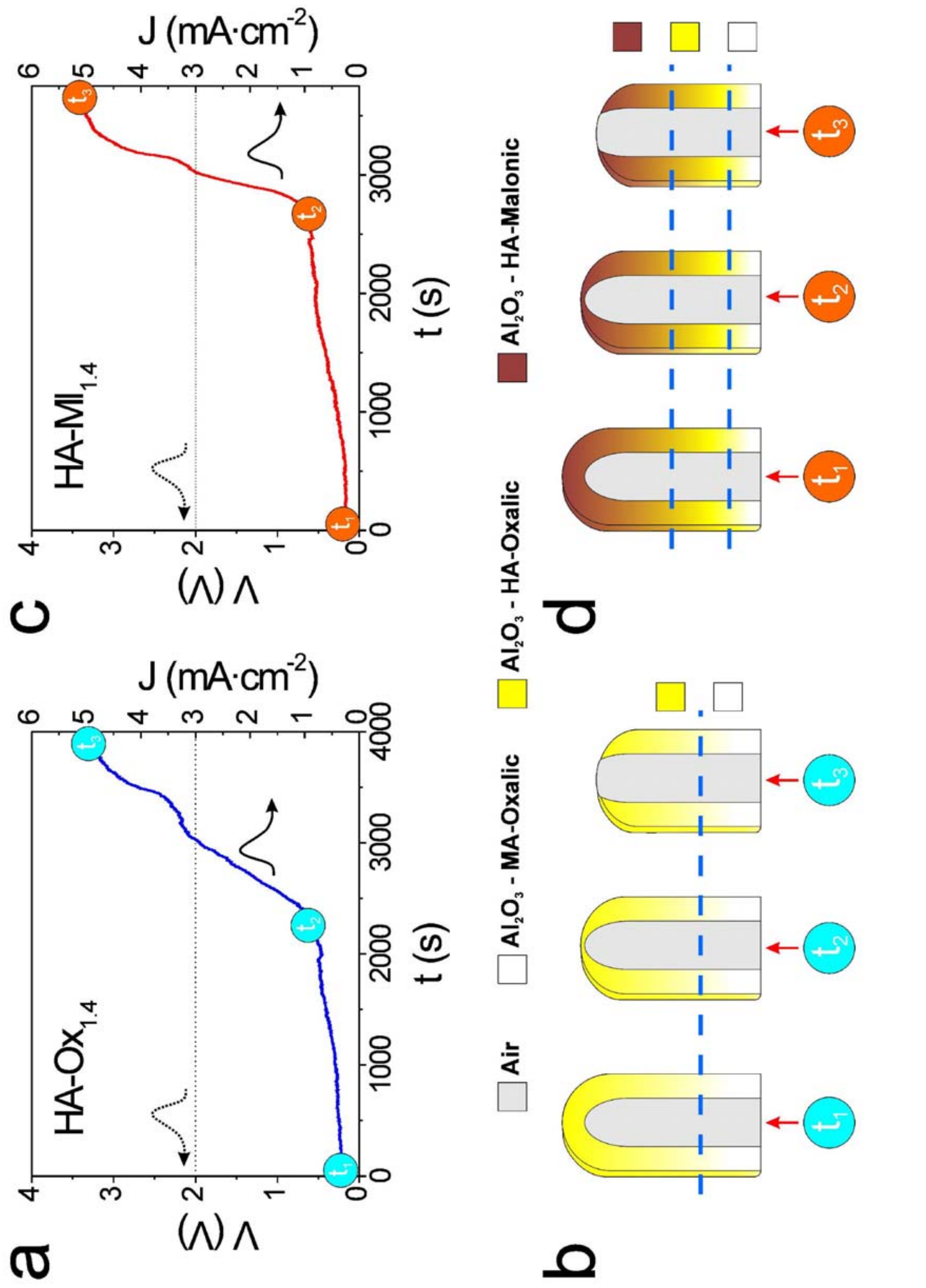

Scheme S2 Voltage and current density-time (*J*-*t*) transient recorded during the pore opening process and illustrations of the pore structure related to each stage of this process for a NAAM fabricated in oxalic ((a) and (b)) and in malonic ((c) and (d)) acids.

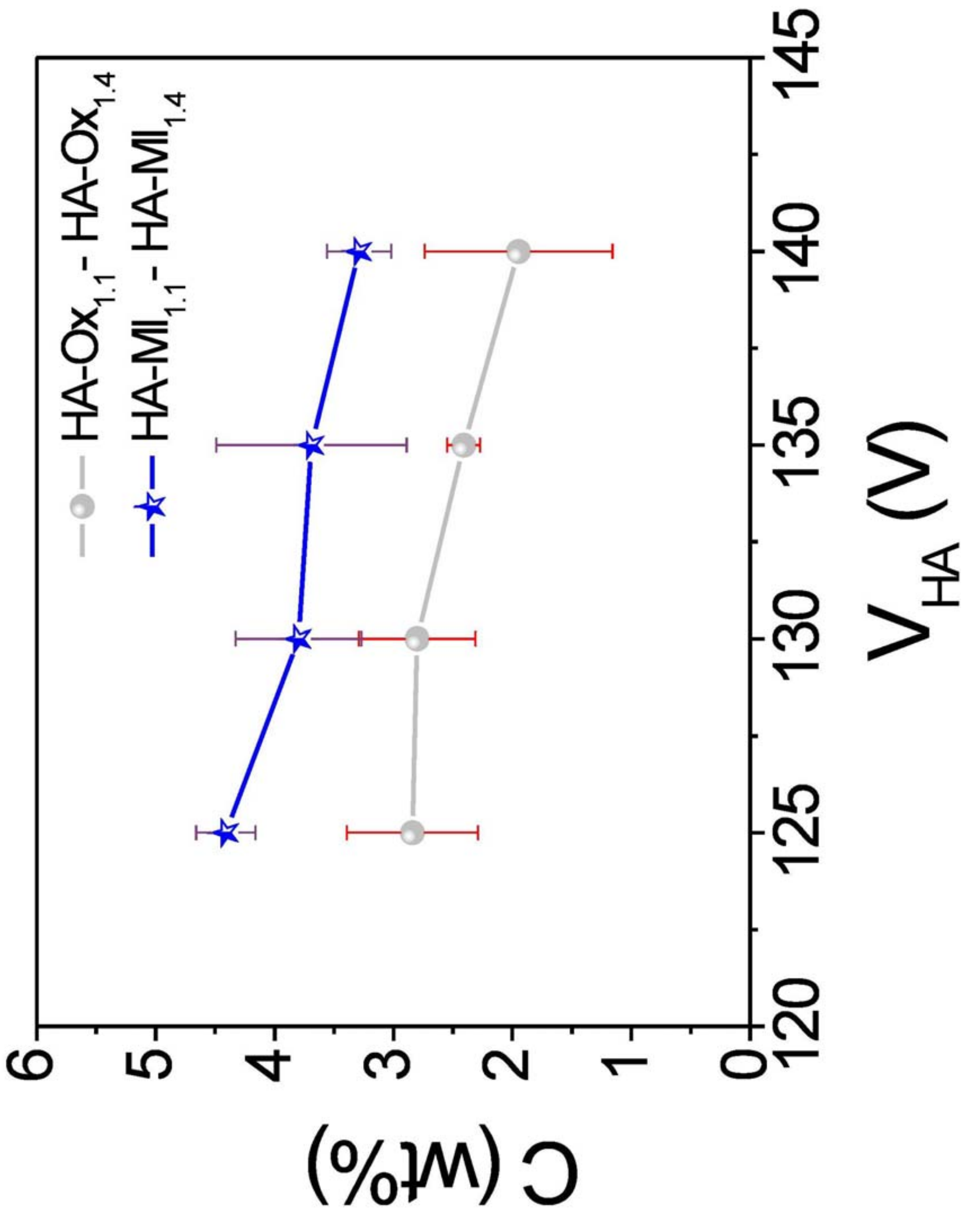

Figure S1 Carbon content of samples HA-$Ox_{1.1}$-HA-$Ox_{1.4}$ and HA-$Ml_{1.1}$-HA-$Ml_{1.4}$ as a function of the hard anodization voltage (V_{HA}) after EDXS analysis.

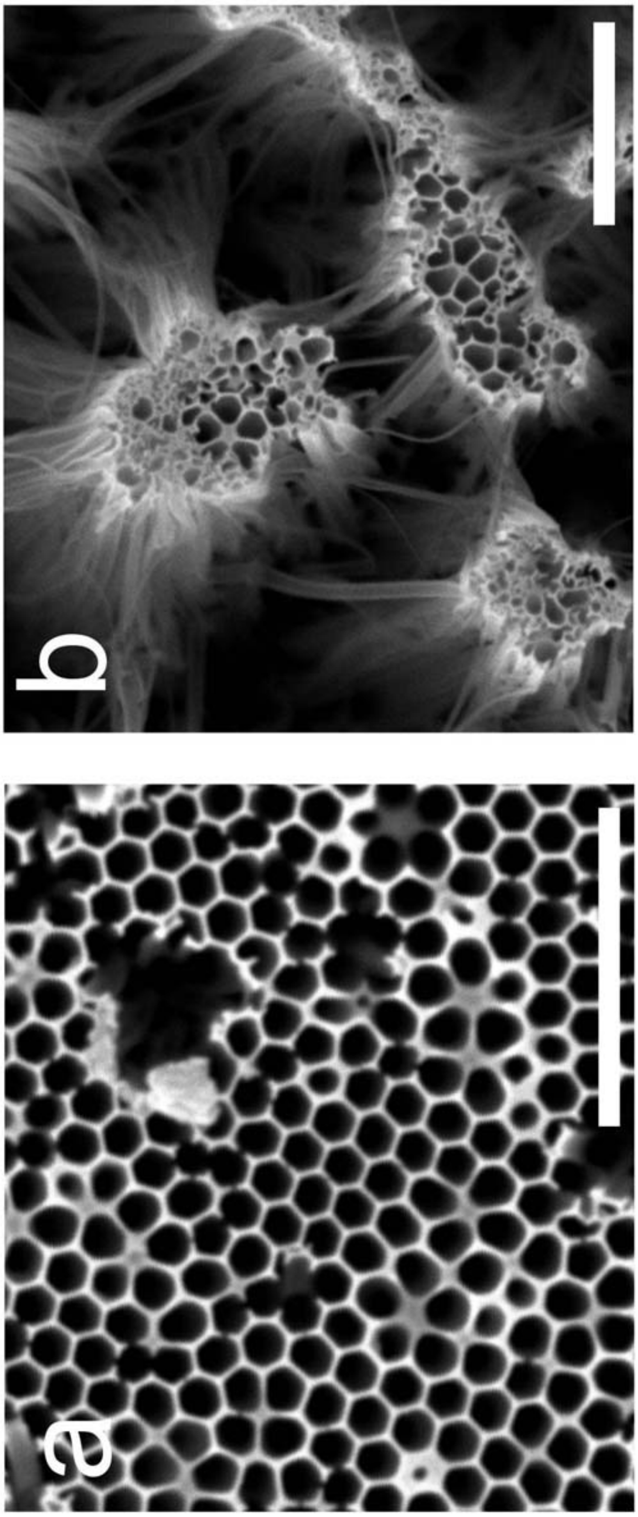

Figure S2 Collapse of NAAMs fabricated in malonic acid after 30 ((a)) and 45 ((b)) min of pore widening in H_3PO_4 5 wt% at 35ºC (scale bar = 2 μm).

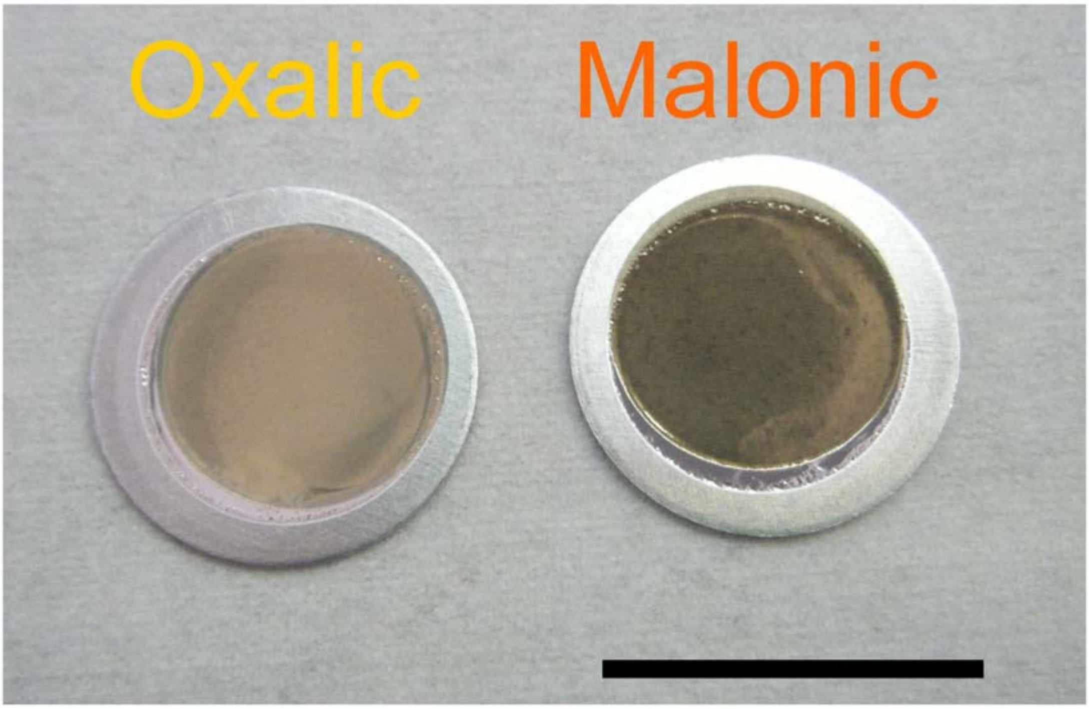

Figure S3 Digital photography of two NAAMs fabricated in oxalic and malonic acid (scale bar = 20 mm).

Sample	V_{HA} (V)	Al (%wt)	O (%wt)	C (%wt)
HA-Ox$_{1.1}$	125	45.36 ± 0.59	51.80 ± 0.21	2.84 ± 0.55
HA-Ox$_{1.2}$	130	45.67 ± 0.45	51.53 ± 0.23	2.80 ± 0.49
HA-Ox$_{1.3}$	135	46.05 ± 0.21	51.54 ± 0.09	2.41 ± 0.14
HA-Ox$_{1.4}$	140	46.35 ± 0.67	51.70 ± 0.13	1.95 ± 0.79
HA-Ml$_{1.1}$	125	43.23 ± 0.09	52.36 ± 0.34	4.41 ± 0.25
HA-Ml$_{1.2}$	130	43.66 ± 0.78	52.54 ± 0.25	3.80 ± 0.53
HA-Ml$_{1.3}$	135	44.08 ± 0.65	52.23 ± 0.22	3.69 ± 0.80
HA-Ml$_{1.4}$	140	44.18 ± 0.11	52.56 ± 0.35	3.29 ± 0.27

Table S1 Average percentage content of Aluminium (Al), Oxygen (O) and carbon (C) of samples HA-Ox$_{1.1}$-HA-Ox$_{1.4}$ and HA-Ml$_{1.1}$-HA-Ml$_{1.4}$ after EDXS analysis. The elemental qualitative analysis was performed on the HA side of each NAAM at three different areas.
